# Keystroke Dynamics Patterns While Writing Positive and Negative Opinions

**DOI:** 10.3390/s21175963

**Published:** 2021-09-06

**Authors:** Agata Kołakowska, Agnieszka Landowska

**Affiliations:** Faculty of Electronics, Telecommunications and Informatics, Gdańsk University of Technology, 80-233 Gdańsk, Poland; nailie@eti.pg.edu.pl

**Keywords:** opinion mining, emotion recognition, behavioural patterns, keystroke dynamics, affect analysis

## Abstract

This paper deals with analysis of behavioural patterns in human–computer interaction. In the study, keystroke dynamics were analysed while participants were writing positive and negative opinions. A semi-experiment with 50 participants was performed. The participants were asked to recall the most negative and positive learning experiences (subject and teacher) and write an opinion about it. Keystroke dynamics were captured and over 50 diverse features were calculated and checked against the ability to differentiate positive and negative opinions. Moreover, classification of opinions was performed providing accuracy slightly above the random guess level. The second classification approach used self-report labels of pleasure and arousal and showed more accurate results. The study confirmed that it was possible to recognize positive and negative opinions from the keystroke patterns with accuracy above the random guess; however, combination with other modalities might produce more accurate results.

## 1. Introduction

This paper deals with analysis of behavioural patterns in human–computer interaction (HCI). Behavioural biometric features are used in security systems or identification applications along with physiological characteristics such as face, palm, fingerprint, or iris images. Among behavioural patterns in HCI an interesting field of study concerns keystroke dynamics and mouse movements as a source of information about a person. As biometric features are stable over time, behavioural patterns may vary depending on disposition of the day or even moment of the day. Among the aspects that influence momentary human behaviour are emotional states. Analyzing behavioural patterns from the perspective of human identification, the point of interest is to find stable patterns and eventually deviations from them. An alternative approach is to analyze variability of the patterns from the perspective of finding indicators of human state. In this paper, we focus on the latter approach and we analyse specifically keystroke dynamics patterns. The advantage of the keystroke dynamics or mouse movements is that they are natural in HCI and do not require any special hardware. Moreover, they are not as intrusive as some other methods [1]. It is possible to record the keyboard and mouse parameters during the usual computer usage.

This paper describes a study in which we analyse keystroke dynamics patterns while writing opinions. The participants were asked to write opinions on their worst and best learning experience, while we captured keystrokes. The research question of the study might be given as follows: Is there any difference in keystroke dynamics patterns while writing positive and negative opinions? We have not found any previous study addressing this aspect. Keystroke dynamics have often been analyzed in order to authenticate users, recognize emotions, or monitor mood. Although emotion recognition seems to be close to our application, it is not the case. The type of opinion does not have to elicit a given emotional state.

The paper is organized as follows: after introduction, related work is summarized in Section 2. Section 3 provides information on the design of the experimental study and methods used for analysing keystroke dynamics, including the definition of metrics characterizing those. Section 4 provides experiment results that is followed by a discussion in Section 5. The main implications of the study and future works are outlined in Section 6.

## 2. Related Work

The research that most relates to the presented study includes works on the quantification of keystroke dynamics and their usage in the analysis of a human state. It falls into the category of behavioural biometrics, relying on the way humans perform some actions, which vary due to different skills, styles, preferences, knowledge or strategy [2]. Behavioural biometrics taken via standard input devices, for example, keystroke dynamics, mouse movements and touch screen gestures, have some advantages. They do not require any special hardware and are unobtrusive for users, so may be recorded during users’ everyday activities without disturbing them. On the other hand, it should be noted that they are not stable over time, which results in a lower accuracy of recognition systems based on these measurements than it is in the case of physiological parameters.

Biometric methods based on keystroke dynamics may be applied in several areas, such as user authentication [3,4,5,6], emotion recognition [1,7,8,9,10,11,12], monitoring mood disorders [13,14] and so forth. The proposed solutions are usually based on hand-crafted features extracted on the basis of keystroke timing or frequency characteristics, for example, dwell time, flight time, typing speed, frequency of using selected keys and so forth.

One of the studies on recognizing emotions has been presented in [9], where some emotional states, that is, confidence, hesitance, nervousness, relaxation, sadness and tiredness, have been recognised with accuracy rates between 77.4% and 87.8% by applying decision trees. In this case, data were gathered during users’ typical activities, such as for example writing messages or using a word processor, but users were also asked to retype a fixed text. Another real-life experiment was described in [10], where only free texts were recorded. In this case a set of timing features were calculated for the most frequent 20 digraphs and 20 trigraphs constituting whole words in Polish. These words were selected on the basis of a frequency dictionary for the Polish language. The obtained accuracies varied from 73% to 87% depending on the participant and emotional state. The study also confirmed the idea that personalised models trained for a selected user to detect one emotional state on the basis of her data give higher results than universal classifiers for all users or multiclass classifiers able to recognise several emotions.

Depending on the application one may try to recognize predefined emotional states but it is also possible to reduce the problem to the recognition of positive vs. negative states as, for example, in [8]. In the mentioned study, it was possible to achieve an accuracy of 89.02% for negative and 88.88% for positive states. It was also shown that typing speed decreased in the case of negative emotions. Other interesting observations on the correlation between emotions and the way of typing have been shown in [15]. The presented study revealed that pleasure correlated with more careful writing, which was demonstrated, for example by using punctuation marks, capitalization and deletion in contrast to fast and careless writing shown in the case of confusion or frustration.

The effectiveness of emotion recognition based on the analysis of keystroke dynamics may be improved if other input modalities are also taken into account. In [16], data collected via both keyboard and mouse are used to infer the boredom and frustrations of a tutoring system users achieving accuracies over 70%. Another example of combining keyboard and mouse data to predict the level of valence and arousal data was presented in [17].

The keystroke dynamics approach may also be implemented on mobile phones, which, besides the virtual keyboard, offer the possibility of incorporating various other sensors to read data at the moment of typing, for example, touch screen or accelerometer [18,19]. A combination of keystroke parameters and physical characteristics, such as heartbeat, motion, energy and sleep, gathered via a smartwatch, was used to predict users’ moods [20].

An interesting approach was presented in [21], where keystroke based stress analysis was combined with a sentiment analysis module and was applied to detect negative messages in social media while they are being written. The combination improved the effectiveness of a system warning about the possibility of propagating high stress or negative replies in network.

## 3. Research Methods

The thesis of this paper might be given as follows: it is possible to recognize pleasure of opinion based on keystroke dynamics patterns. Based on the presented related work, there has been no other similar study. This section provides a description of the methods that were applied in the study design, execution, and the post-processing of the data.

### 3.1. Experiment Design

To verify the research hypothesis, a semi-experiment was designed and conducted with human participants. The study was performed in a laboratory setting at our university. Full randomization of subject selection was not possible in a laboratory setting located in one place only, therefore a group of convenience was used (students of the university). The consequences of such a choice are discussed in Section 5. The students were volunteers recruited from one academic year. A single group within-subject design was used as we wanted to identify the difference between the two conditions of the same person (and not the individual differences).

The outline of the single participation scenario was as follows. First, the keystroke capturing software was launched. The subject was asked to fill in a multi-page questionnaire, including metric data, opinion on the best learning subject he/she participated in, opinion on the worst learning subject, opinion on the best teacher, opinion on the worst teacher. In between writing opinions students additionally filled in emotion-related questionnaires and noted down local computer time. Then the keystroke capturing software was turned off and the raw keystroke data were saved as a file. Writing down the local computer time was required as the keystroke software used timestamps based on that. In further analysis we were able to cut the keystroke time series to the parts assigned to each of the four opinions.

**Figure 1:**
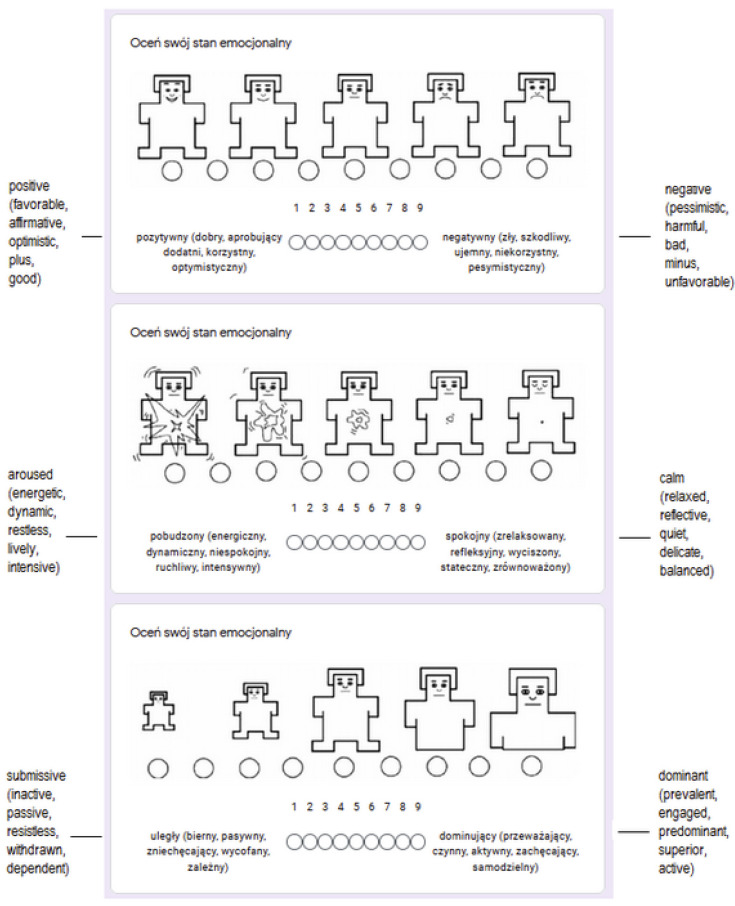
Emotional state self-assessment scale—a screenshot with adjective translations.

The questionnaire used for capturing the emotional state of participants before and between writing opinions was Self-Assessment Manikin (SAM) [22], using a 9-point scale along with the visual representation. The screenshot of the adapted SAM questionnaire used is provided in Figure 1.

We have decided to use the SAM scale as it was connected to the purpose of this study. As the main goal was to capture differences between positive and negative opinions, the pleasure of emotional state was of the primary interest. Therefore, we have decided to capture emotions with the three-dimensional PAD (pleasure-arousal-dominance) scale and the SAM questionnaire is the one supporting it. The SAM scale might also cause some confusion, when left undescribed. The dominance dimension is problematic for some to understand. In order to overcome this obstacle, we have used SAM visual representation accompanied with some adjectives describing extreme values of the scale.

### 3.2. Experiment Execution

The experiment was conducted in the laboratory setting at the university premises. The computer stands were standardized—the same computer type, keyboard and mouse were used. The participants were recruited from students of the computer science course; the students were not paid for their participation, but they were offered an extra exam date to select apart from the standard one available to every student during the examination session. There were 50 students who took part in the study (40 males, 10 females, age mean 21 ± 1 years). We wanted the sample to be as homogenous as possible, as we did not want to analyze the influence of age on keystroke patterns, considering it as a confounding variable. Differences in keystroke dynamics among various age groups have been investigated in a number of research studies focusing on recognizing age on the basis of keystroke dynamics [23,24,25]. The data were anonymized. One student’s data were excluded from further analysis, so for the analysis we took data from 49 participants. The reason for the exclusion was that the student entered random letters instead of the opinions he was asked for. While students were writing opinions, the keystroke data were captured using an original program. The program was turned on before the student started writing the opinion, and was turned off after the sequence of opinions was finished. A single opinion writing session was planned for 15 min (duration time mean: 13′22, min: 6′09, max: 21′11). The raw keystrokes’ time series were then processed as described in Section 3.3.

**Figure 2:**
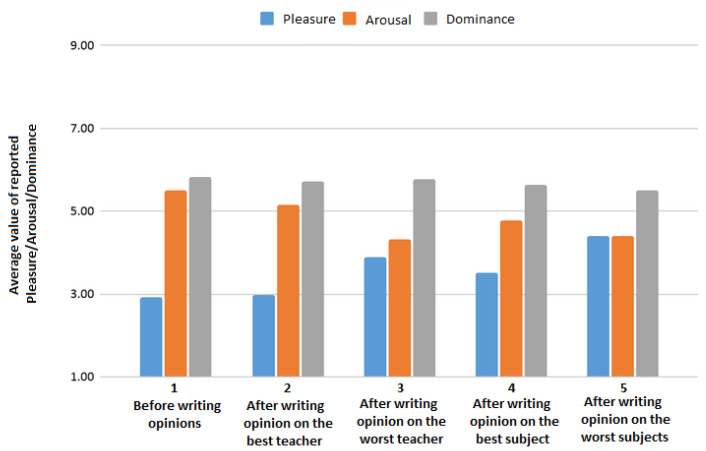
Average values of pleasure, arousal and dominance reported at different stages of the experiment session.

At the beginning of the experiment, and after writing each of four opinions, a participant filled a self-report as described in Section 3.1. Figure 2 presents mean values of pleasure, arousal and dominance calculated on the basis of five reports from all participants. It shows how the values change over time. In the case of pleasure and arousal, some variations may be observed. Dominance seems to be the most stable over time. A part of this study is the analysis of a possible relationship between these changes of PAD values depending on the type of opinion (positive/negative).

**Figure 3:**
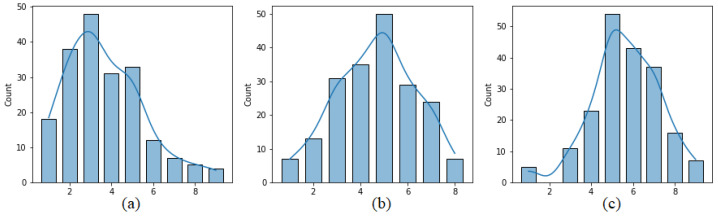
Distribution of (**a**) pleasure, (**b**) arousal and (**c**) dominance values.

Figure 3 presents the distribution of pleasure, arousal and dominance values calculated on the basis of the four reports from each user sent after writing the four opinions. It can be seen that pleasure values are moved toward positive values. In the case of arousal the extreme value of nine, indicating the lowest level of arousal was never reported.

**Figure 4:**
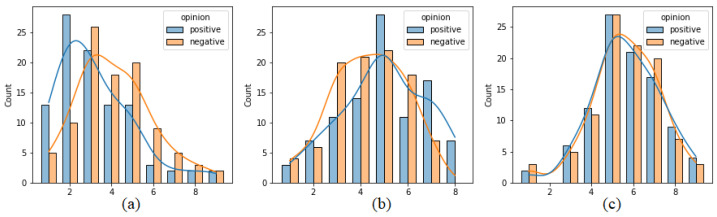
Distribution of (**a**) pleasure, (**b**) arousal and (**c**) dominance values, separately for positive and negative opinions.

Figure 4 presents analogous histograms but generated separately on the basis of reports sent after positive and negative opinions. Some differences in the distributions may be observed in the case of pleasure and arousal. Pleasure values reported for positive opinions are moved toward lower values (indicating positive affect) more than in the case of negative opinions. The two distributions for dominance almost overlap, which may suggest that dominance values do not differ between reports after positive and negative opinions. To actually compare these distributions, a statistical test was used. Due to the fact that the values originate from the ordinal 9-point Likert scale, the non-parametric Mann–Whitney test was applied. The results are presented in Table 1. It can be seen that the distributions of labels connected with positive and negative opinions are significantly different (*p*-value < 0.05) in the case of pleasure and arousal.

**Figure 5:**
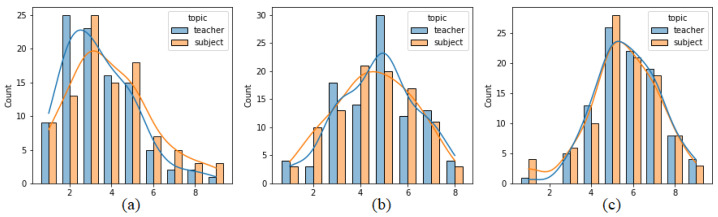
Distribution of (**a**) pleasure, (**b**) arousal and (**c**) dominance values, separately for teachers and subjects.

Figure 5 presents analogous histograms created separately on the basis of reports sent after opinions on teachers and subjects. In this case, there is also some discrepancy between the two distributions for pleasure, where opinions on teachers are assigned more negative values than on subjects. The results obtained by applying the Mann–Whitney test are presented in Table 2. The distributions of teacher and subject labels are significantly different (*p*-value < 0.05) in the case of pleasure.

### 3.3. Keystroke Dynamics Feature Extraction

The process of feature extraction performed in this study was performed on the basis of a procedure from our earlier study presented in [11] with some slight modifications. The first stage of data processing was segmentation. Due to the fact that no user types continually, the whole sequence of keystrokes was split into a series of shorter sequences depending on the presence of pauses. To identify the limits of typing sequences an idle threshold was introduced. If the time between depressing a key and pressing the next one exceeded the idle threshold, then the split was made. The greater the value of the threshold, the longer keystroke sequences were extracted. All timing characteristics described later in this section were calculated regarding the extracted partial sequences. The extraction was performed for the threshold value of 3 s.

After segmenting the data, a feature extraction procedure was performed. A number of parameters were calculated on the basis of raw data. They may be divided into the following groups: digraph features, trigraph features, special digraph features, frequency features and typing speed. The total number of parameters was 51. The detailed list of all features is presented in Table 3.

Digraph and trigraph features are timing characteristics for two-key and three-key sequences. They are all based on parameters commonly used in keystroke dynamics analysis, that is, the time a key is pressed, the time between releasing a key and pressing the next one, the duration of key sequences (the time between pressing the first and depressing the last key in a sequence), and the times between subsequent key presses. Moreover, the number of events for a digraph or trigraph was also calculated. These are the numbers of all key down and key up events in a sequence, so it is usually four for a digraph and six for a trigraph. Sometimes, especially when a user types quickly, it happens that a user presses the next key before depressing one. In such cases, additional events may appear between those coming from a graph and then the values for these attributes may differ from four or six. A data sample from a user contains many digraphs and trigraphs. The parameters were calculated for all of them and then their mean values and standard deviations were saved as feature values in a feature vector representing the sample.

Some digraphs have been treated as special sequences in the case of this application. These are digraphs containing either the left or right shift key as the first one. Therefore some digraph parameters were calculated for digraphs starting from the left and the right shift.

Another group of features are frequency parameters. In contrast to digraphs and trigraphs, they do not describe keystroke rhythm. Some of the parameters may indicate the way users make corrections (the use of backspace, delete keys), move across the text (pgup, pgdn, home, end, up, down, left, right) or take care of punctuation. The frequency was calculated as the number of a selected symbol to the total number of keystrokes. One of the frequency features was calculated in a different way, that is, the number of capital letters to the total number of letters.

Finally, the typing speed, which indicates the number of keystrokes per second, was calculated.

### 3.4. Data Preprocessing

The classification experiments were performed both for original feature values and the values obtained after some normalisation. Several normalisation procedures were applied to the extracted features. For each user, five feature vectors were extracted. The first one was a baseline vector. This vector contained features obtained on the basis of the whole text typed by a user, that is, the whole session was not divided into positive and negative parts but treated as a single typing phase. The other four vectors were extracted on the basis of two positive and two negative pieces of text, respectively. Then two types of training sets were created:

- absolute data set containing the original four vectors form each user;
- relative data set containing for each user the four vectors after subtracting the user’s baseline vector from them.

Moreover, both sets were normalized by standardising them to have zero mean and the standard deviation of 1.

### 3.5. Analysis Methods

Data analysis was conducted in two main stages. The aim of the first stage was to evaluate the proposed features from the point of view of their discriminative power. First of all it was verified whether the values of the keystroke patterns differ significantly between positive and negative opinions. Moreover, a mutual information criterion was used to evaluate the dependency between features and classes for different classification tasks, that is, positive vs. negative opinions, high vs. low level of pleasure, high vs. low level of arousal. Mutual information is often used in feature selection as a measure of the degree of relatedness between datasets has been applied [26].

The aim of the second stage was to train and test classifiers for these three classification problems. Several classifiers were trained and tested. In the case of recognising the level of pleasure or arousal three different labeling procedures were applied depending on a threshold value. The detailed description of the performed analysis and the obtained results are presented in the next section.

## 4. Experiment Results

### 4.1. Feature Evaluation

The proposed set of hand-crafted features contains 51 parameters. Most of them have been already incorporated in other research studies [9,10,11]. Obviously, not all of them may be equally effective in this task. Therefore it is worth analyzing the importance of individual parameters.

#### 4.1.1. Identifying Features That Differ Significantly between Positive and Negative Opinions

The aim of the first test was to verify which features show significantly different values between positive and negative opinions. Dependent *t*-test for paired samples was used to perform this task [27]. It is defined as follows:

(1) $$t=\frac{\bar{d}}{s_{d}}\sqrt{n-1}$$

where $\bar{d}$ is the mean difference between the values obtained for positive and negative opinions respectively; $s_{d}$ is the standard deviation of the differences; *n* is the number of degrees of freedom, that is, the number of pairs of samples, for which the difference is calculated. In our case a two-tailed test was applied, because no assumption was made on the direction of the observed changes, that is, feature values may either increase or decrease.

The second column of Table 4 presents the test results for all features. The t-statistic exceeded critical value for the significance level *p* = 0.05 for 12 features, which are marked bold. Most of them are timing characteristics describing digraphs and trigraphs. One of the features belongs to the frequency parameters and it describes the frequency of using spacebar. Eventually, typing speed turned out to have significantly different values between the positive and negative opinions.

**Figure 6:**
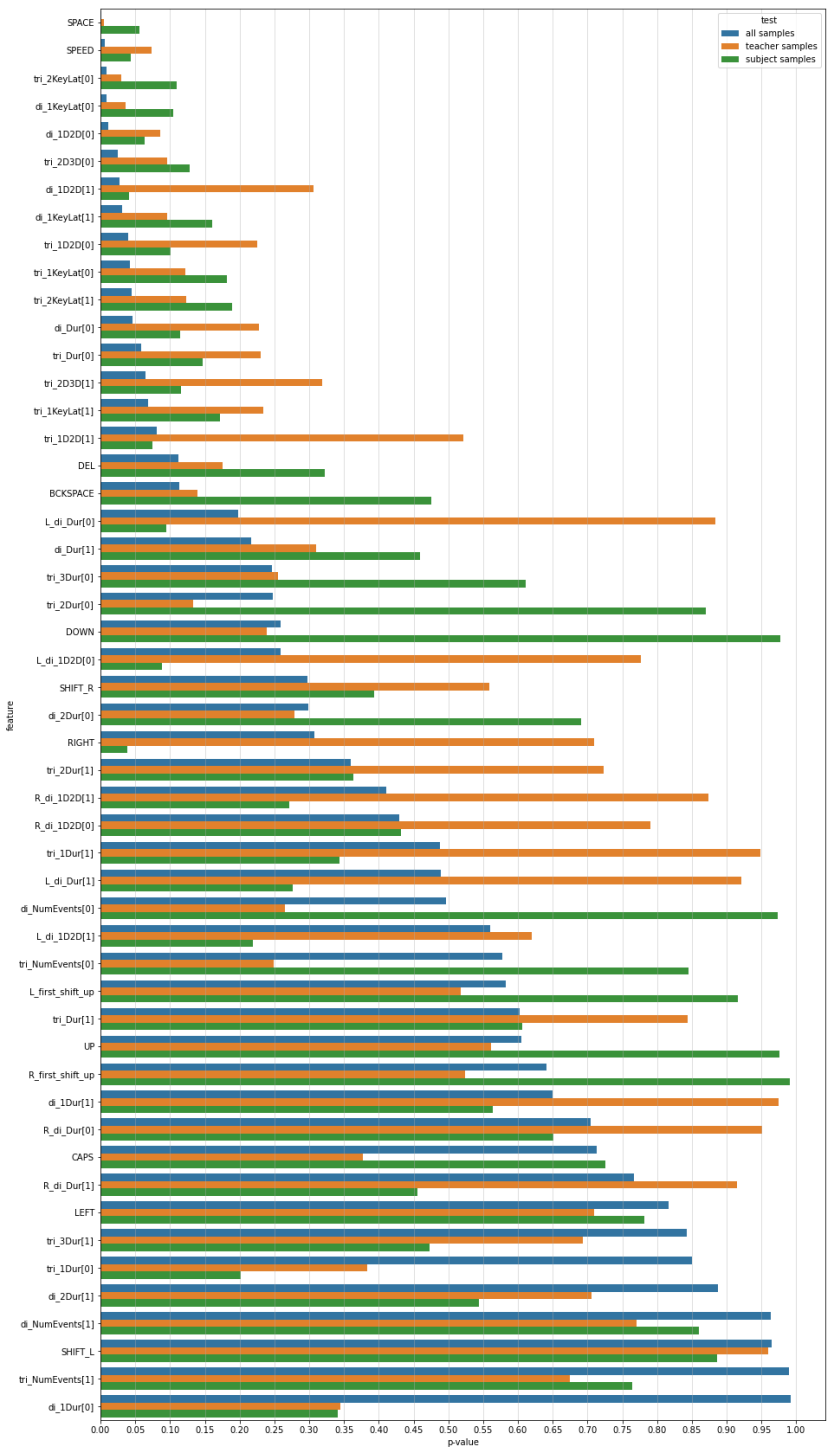
*p*-values obtained after applying paired *t*-test for positive/negative opinions on the basis of three sets of samples. Features are sorted according to the results obtained on the basis of a dataset containing all samples.

The other two columns of Table 4 present the results of the same test calculated on the basis of opinions on teachers or subjects, respectively. The values are obviously higher, due to a lower number of samples. In each case there are three features for which the test exceeded critical value for the significance level of 0.05. The results are also presented on a bar plot where features are sorted according to increasing *p*-values obtained for the dataset containing all samples (Figure 6).

**Figure 7:**
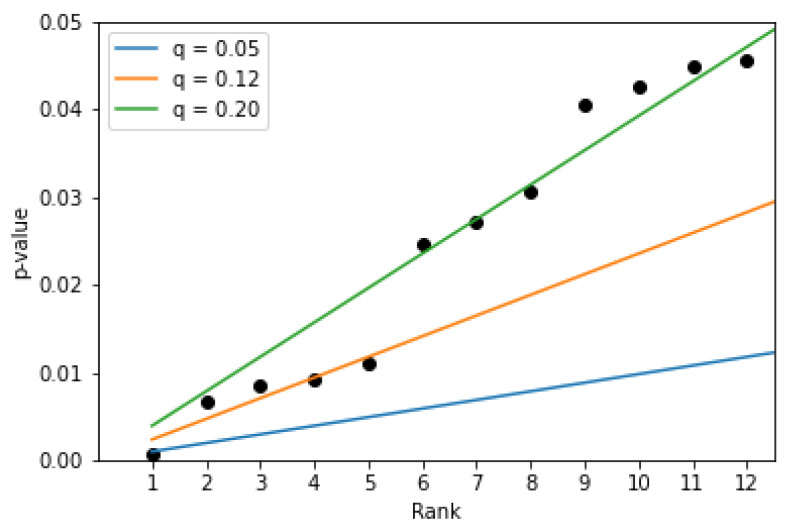
Top 12 *p*-values obtained after applying paired *t*-test for positive/negative opinions on the basis of all samples and the cutoff lines set according to the Benjamini–Hochberg procedure.

Testing the set of *n* features is the multiple testing problem, which means that on average αn features are falsely recognized as significant, where α is the significance level. To prevent inflation of a type-I error it is possible to apply a procedure which adjusts the *p*-values. One of these methods is the Benjamini–Hochberg (BH) procedure, which allows control of the false-discovery rate (FDR) defined as the expected proportion of type-I errors among the rejected hypotheses [28]. It requires sorting the *p*-values, then finding the largest *p*-value lower than qr/n, where *r* is the rank of a *p*-value in the sorted list, *q* is the level at which the FDR is controlled. According to the procedure, the null hypotheses for the *p*-values up to the identified one and including this one are rejected. Figure 7 presents 12 lowest *p*-values and cutoff lines set according to the BH procedure for different values of *q*, which controls the level of FDR. It can be seen that if we set the level to 0.05 (blue line) only one feature will be selected as a parameter with values significantly different between positive and negative opinions. This is the SPACE feature. For the level equal to 0.12 (orange line), five features are identified. To identify the 12 features, which were selected without applying the BH procedure, one would have to set the level *q* to 0.2 (green line), which means that the expected values of features falsely identified as significant would be 0.2. Applying the BH procedure for the other two sets of samples, that is, for opinions only on teachers or only on subjects, did not reveal features with values that were significantly different between positive and negative opinions for the mentioned levels of controlling FDR.

#### 4.1.2. Estimating Mutual Information

The aim of this test was to measure the dependency between feature values and the labels. Depending on various criteria, several label assignments of data samples were taken into account in this experiment:

- type of opinion, either positive or negative, assigned according to the opinions the participants were asked to write;
- low (greater than 5) or high (lower than 5) pleasure depending on values from the self-report, samples with pleasure values equal to 5 were removed from the dataset;
- low (greater than 5) or high (lower than 5) arousal depending on values from the self-report, samples with arousal equal to 5 were removed from the dataset.

**Figure 8:**
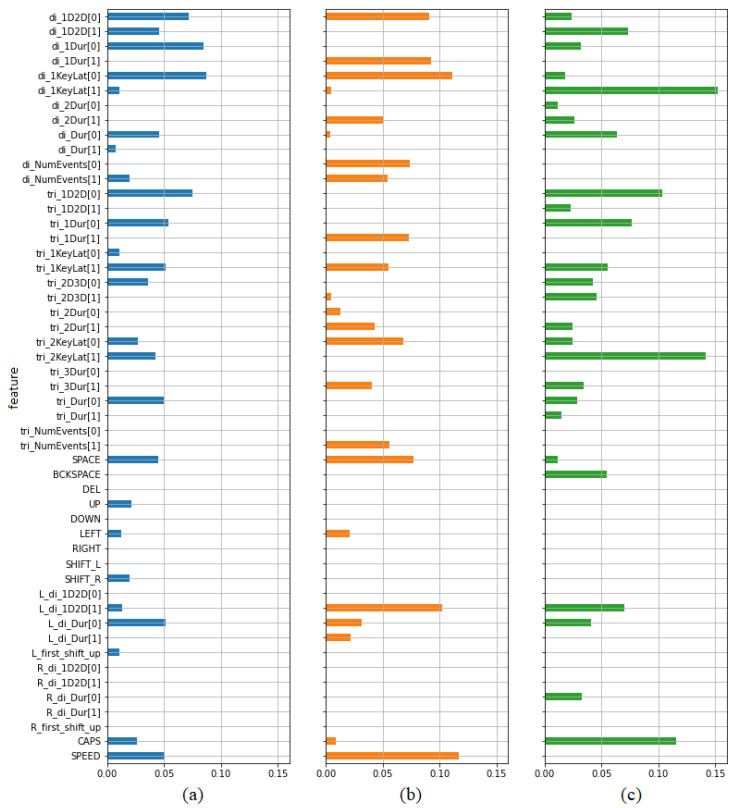
Mutual information values indicating the dependency between features and labels in the task of discriminating between positive and negative opinions, calculated for (**a**) all samples, (**b**) teacher samples, (**c**) subject samples.

Table 5 presents the calculated values of mutual information. Higher values indicate greater dependency. The first three columns contain values indicating features’ ability to predict the type of opinion (positive/negative) calculated separately for the whole data set (column 1), subset of samples from opinions on teachers (column 2) and subset of samples from opinions on subjects (column 3). It has been also presented on bar plots (Figure 8). In each case a set of the best predictors may be indicated. Most of them are digraph and trigraph parameters as it was in the case of previously described paired *t*-test. Most features selected in this way have been also selected using the previous test. However, there are several parameters showing some predictive power from the point of view of one criterion, but not from the other. From the set of frequency features only the frequency of using spacebar seems to be worth taking into account. Both criteria indicate typing speed as a potentially valuable predictor.

**Figure 9:**
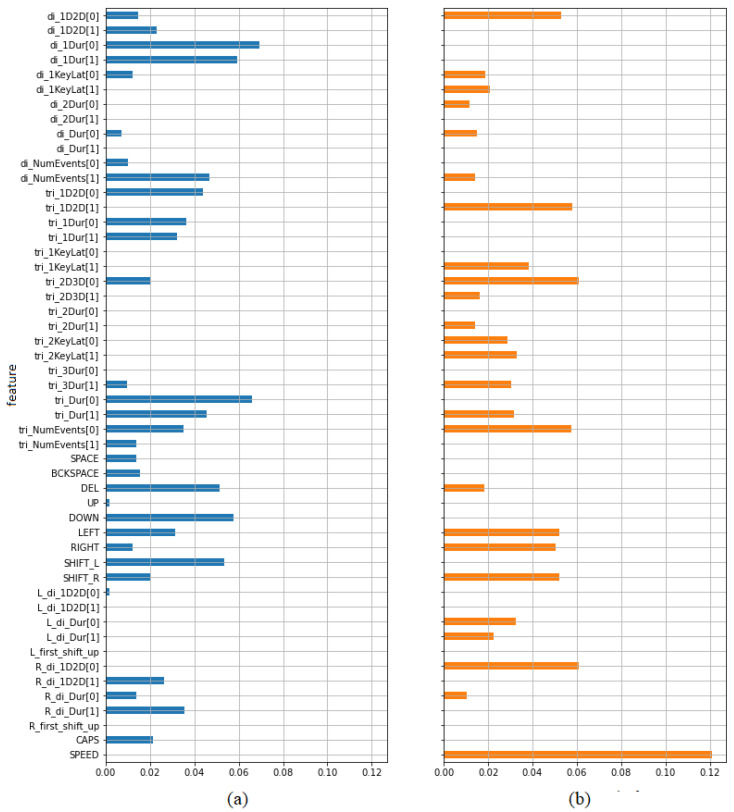
Mutual information values indicating the dependency between features and labels in the task of discriminating between high and low level of (**a**) pleasure, (**b**) arousal.

The last two columns of Table 5 present the effectiveness of the features in discriminating between high and low pleasure and arousal respectively. It has been also presented using bar plots (Figure 9). It can be seen that typing speed is especially worth taking into account as a predictor of arousal.

### 4.2. Estimating the Significance of Differences between PAD Labels for Positive and Negative Opinions

The aim of this test was to verify whether the label values of pleasure, arousal and dominance reported by the participants after the positive and negative opinions were significantly different. Although it has been already shown in Table 1 that the distributions of pleasure and arousal labels differ significantly between positive and negative opinions, it is also possible to look at these two data samples as dependent ones. The opinions may paired, that is, each positive opinion on a topic may be accompanied by a negative opinion on the same topic written by the same person. From this point of view, it is worth verifying whether the reported labels change significantly after changing the type of opinion. In order to verify this, the Wilcoxon signed-rank test was applied. It is a non-parametric equivalent of the *t*-test for paired samples.

Table 6 presents the *p*-values obtained after applying the two-sided Wilcoxon test for each of the three PAD dimensions. It shows that in the case of pleasure and arousal the differences between positive and negative labels are significant (*p*-value < 0.05). No significant differences between positive and negative labels were observed for dominance.

### 4.3. Classification

Three classification problems were taken into account during the tests. The first one was to recognize whether an opinion is positive or negative. The other two problems were training classifiers for pleasure and arousal, respectively. Several classifiers, that is, SVM, random forest, naive Bayes and k nearest neigbours, have been applied and tested. The results obtained using the SVM classifier outperformed other ones. Therefore the following subsections present results obtained using SVM. Because of the high number of features when compared to the number of samples, the dimension has been reduced by removing features with very low variance and then by removing highly correlated attributes. In each case classifiers were trained for various sets of data, that is, either absolute or relative as it was described in Section 3.4, either scaled or not, either after reducing the number of features or not. The experiments do not show high impact of scaling and reducing the number of parameters. All tables in the following subsections present the results obtained for unscaled feature values, reduced number of features, both for absolute and relative datasets.

#### 4.3.1. Recognizing Positive vs. Negative Opinions

The aim of the first classification experiments was to verify whether it was possible to recognize if an opinion was positive or negative on the basis of keystroke dynamics. To train this classifier a training set containing 196 samples was created. The labels were assigned according to the opinions the participants were asked to write. There were 98 samples for each of the two classes. Forty nine opinions on the best teacher and 49 on the best subject were labeled as positive. Negative labels were assigned to the opinions on the worst teacher and the worst subject. The PAD labels for SAM questionnaire were not taken into account in this case. Table 7 presents the results obtained by applying an SVM classifier trained and tested in a 10-fold cross validation procedure. The parameters of the SVM model were adjusted in a grid search procedure. It turned out that the results obtained for relative feature values were better than for the absolute ones. The average values of precision, recall and F1 measurements were around 0.62.

#### 4.3.2. Pleasure and Arousal Recognition

The aim of these experiments was to recognize the level of pleasure or arousal. Due to the small number of samples and the fact that some levels from the 9-point scales were scarce in the collected date, the problem was reduced to a binary task. The levels were merged to form two classes representing High or Low level. The different merging procedure were implemented, depending on the setting of the threshold value on the 9-point scale.

- L1: samples labeled with values greater than 5 were assigned Low level, samples labeled with values lower than 5 were assigned High level, samples labeled with 5 were removed from the data set;
- L2: samples labeled with values greater or equal to 5 were assigned Low level, samples labeled with values lower than 5 were assigned High level;
- L3: samples labeled with values greater than 5 were assigned Low level, samples labeled with values lower or equal to 5 were assigned High level.

The presented merging procedures resulted in different training sets with different class distributions as shown in Table 8. In some cases, the obtained datasets were highly imbalanced, which may have a disadvantageous influence on classifiers’ efficiency.

Table 9 and Table 10 present classification results obtained after training the SVM classifier to recognize the level of pleasure and arousal, respectively. In each case the models were trained and tested in a 10-fold cross validation procedure. The parameters of the SVM model were adjusted in a grid search procedure. As it was in the case of recognizing positive/negative opinions, the results obtained for the relative data set are usually better than for the absolute one, but they differ much between the data sets created using different labeling approaches. High class imbalance made the results for the minority class, that is, the class of Low levels of pleasure or arousal, lower in each case. In the case of pleasure the best average results were obtained for L3 labeling, where the weighted average of F1-score was 0.76. However, it should be noted that the results for Low class, both precision and recall, are unacceptably low in this case. In the case of arousal L1 and L3 labeling procedures lead to F1-score of around 0.65. The training data set created using the L2 labeling did not let train an arousal classifier assigning all samples to one class. Therefore the results for this labeling method were not presented in Table 10.

## 5. Summary of Results and Discussion

In this study we captured keystroke dynamics patterns while writing positive and negative opinions. The patterns were quantified as 51 features and then classification was performed with labels of positive/negative opinions as well as labels of self-reported pleasure and arousal.

The results of the study in terms of comparison between the different keystroke patterns (features) might be summarized as follows:

- based on t-Student test (with 0.05 *p*-value threshold) 12 out of 51 features show significant differences between positive and negative opinions, including five digraph features, five trigraph features, frequency of using spacebar and typing speed, but only one feature after applying the Benjamini–Hochberg correction with control of false discovery rate at the level of 0.05;
- based on mutual information measure top eight features (mutual information > 0.05) might be indicated in distinguishing between positive and negative opinions, that is, three digraph features, three trigraph features, one shift feature and typing speed;
- based on mutual information measure (mutual information > 0.1), one might find the top three features in distinguishing between positive and negative opinions on teachers and the top four features in distinguishing between positive and negative opinions on subjects; however, the features are different for both sets.

To summarize, none of the feature groups (digraph, trigraph, shift, frequency-based) has a dominant representation in the significant features; however, one might find the frequency of using spacebar and typing speed as the two mostly connected with labels. There are alternative features that might be calculated for keystroke dynamics, including for example the timing characteristics calculated for the most common sequences or the most common words in a given language [10]. Apart from mean values and standard deviations of some parameters, one may also take into account other statistics, for example, selected quantiles. Subjective selection of the feature set is among the drawbacks of the study; however, we have covered the most used ones.

The results of the study in terms of classification results might be summarized as follows:

- relative data sets containing vectors normalised by subtracting a baseline vector for each user lead to better results;
- classification of positive and negative opinions was above random guess (with total F1 score exceeding 0.62), but the result is not impressive;
- classification of two pleasure levels was dependent on label merging procedure, with average F1-score of around 0.76 at the best case, but the results for two classes are highly unbalanced showing unacceptable result for the minority class;
- classification of two arousal levels was dependent on label merging procedure, with 2 out of 3 cases showing accuracy above random guess (with average F1-score of around 0.65);
- classification of dominance labels was not performed as no significant differences were found for high and low dominance.

To summarize, it is possible to recognize positive and negative opinions from the keystroke patterns with an accuracy above random guess; however, one must take into account that during the study not all participants writing positive and negative opinions actually felt the emotions connected with them—they were asked to revive the memory of the best/worst learning experience; however, the disposition of the day and temporary mood connected with the experimental setup could also influence the keystroke patterns.

As has been described in Section 4.3.2, the levels of pleasure and arousal were merged and thus the problem was reduced to a binary one. It is well known that people may have various predispositions to selected emotional states, also to certain levels of arousal or pleasure. Therefore, setting the same threshold value for all users to distinguish between low and high levels of pleasure or arousal may not be the right approach. Some personalisation implemented at this stage might lead to better labeling and in turn better performance of the trained classifiers. This idea has been applied in [29] for example, where personalised z-score normalisation was used while transforming from a 5-point scale to binary in the task of boredom detection. Unfortunately, it was not possible in our case because there were only four labeled samples from each user. In this study we tested three different methods of merging labels into two classes, however one might propose a different one.

Please note that all of the reported results are for the SVM classifier. We have tested alternative ones, including random forests, naive Bayes, k nearest neigbours, but none of them produced better results. As only a limited number of classifiers was used, one might propose using different ones.

Among the other validation threats to the study one may point out homogenous participant group. Although the group consisted of 50 people, it was homogenous—only students, aged 20–22 took part in the study. We are aware of the fact that this might lead to limited generalisability of the findings.

## 6. Conclusions

The study provided some preliminary results that indicate that keystroke dynamics patterns might contribute to opinion mining research. However, as the differences in patterns for positive and negative opinions were only slightly different, one might combine the patterns with other modalities. Interesting future studies might include combination of keystroke patterns with mouse patterns or with physiological signals. Sentiment analysis of the opinions of participants might also be performed, which will be one of our future studies. Among the key challenges that are faced by such a study, we would like to emphasize the labeling issue. We used labeling by a predefined task (stimuli) and by self-report; however, both are susceptible to different confounding factors and might not reflect the “ground truth” (i.e., the actual emotional state). Eventually, a future study would also require a larger and less homogenous group of participants to incorporate other variables, such as age, gender, technical skills, typing experience, fatigue and so forth.

The study has several practical implications. Keystroke dynamics patterns might be an interesting modality to include in multi-channel emotion recognition, as they are easy to collect and are an unobtrusive method of monitoring in the human–computer interaction context. There is an issue of privacy in the tracking keystrokes studies, that is, one might input logins and passwords or private messages. The issue must be addressed for ethical reasons in such research and one of the methods, used in this study, does not trace specific letters and digits keys, and registers only general information on pressing a letter key. This study might be interesting for both researchers and practitioners who track human activity on computers in order to recognize human emotional states. 

## Figures and Tables

**Table 1:** Comparing the distribution of PAD values for positive and negative opinions—mean values and results of Mann–Whitney test.

	Pleasure	Arousal	Dominance
positive sample mean	3.24	4.97	5.67
negative sample mean	4.14	4.36	5.63
test statistic	3290.0	3831.5	4796.0
*p*-value	0.0001	0.0065	0.4944

**Table 2:** Comparing the distribution of PAD values for teacher and subject opinions—mean values and results of Mann–Whitney test.

	Pleasure	Arousal	Dominance
teacher sample mean	3.43	4.74	5.74
subject sample mean	3.96	4.58	5.56
test statistic	4038.5	4546.5	4605.0
*p*-value	0.0254	0.2570	0.3067

**Table 3:** Features extracted from raw data. Most features are mean and standard deviation denoted as [0] and [1] respectively.

Feature		Description
Digraph features		
di_1D2D[0],	di_1D2D[1]	duration between the 1st and the 2nd down keys of a digraph
di_1Dur[0],	di_1Dur[1]	duration of the 1st key of a digraph
di_1KeyLat[0],	di_1KeyLat[1]	duration between the 1st key up and the next key down of a digraph
di_2Dur[0],	di_2Dur[1]	duration of the 2nd key of a digraph
di_Dur[0],	di_Dur[1]	duration of the whole digraph from the 1st key down to the last key up
di_NumEvents[0],	di_NumEvents[1]	number of key events for a digraph
Trigraph features		
tri_1D2D[0],	tri_1D2D[1]	duration between the 1st and the 2nd down key of a trigraph
tri_1Dur[0],	tri_1Dur[1]	duration of the 1st key of a trigraph
tri_1KeyLat[0],	tri_1KeyLat[1]	duration between the 1st key up and the next key down of a trigraph
tri_2D3D[0],	tri_2D3D[1]	duration between the 2nd and the 3rd down key of a trigraph
tri_2Dur[0],	tri_2Dur[1]	duration of the 2nd key of a trigraph
tri_2KeyLat[0],	tri_2KeyLat[1]	duration between the 2nd key up and the next key down of a trigraph
tri_3Dur[0],	tri_3Dur[1]	duration of the third key of a trigraph
tri_Dur[0],	tri_Dur[1]	duration of the whole trigraph from the 1st key down to the last key up
tri_NumEvents[0],	tri_NumEvents[1]	number of key events for a trigraph
Shift digraph features		
L_di_1D2D[0],	L_di_1D2D[1]	time between pressing the 1st and the 2nd key in a digraph starting from the left shift
L_di_Dur[0],	L_di_Dur[1]	duration of a digraph starting from the left shift (time between pressing the 1st and releasing the 2nd key)
L_first_shift_up		percentage of time when the left shift starting a digraph is released before releasing the 2nd key
R_di_1D2D[0],	R_di_1D2D[1]	time between pressing the 1st and the 2nd key in a digraph starting from the right shift
R_di_Dur[0],	R_di_Dur[1]	duration of a digraph starting from the right shift (time between pressing the 1st and releasing the 2nd key)
R_first_shift_up		percentage of time when the right shift starting a digraph is released before releasing the 2nd key
Frequency features		
SPACE		frequency of using spacebar
BACKSPACE		frequency of using backspace key
DEL		frequency of using delete key
UP		frequency of using up arrow
DOWN		frequency of using down arrow
LEFT		frequency of using left arrow
RIGHT		frequency of using right arrow
SHIFT_L		frequency of using left shift
SHIFT_R		frequency of using right shift
CAPS		frequency of using caps lock
Typing speed		
SPEED		average number of keystrokes per second

**Table 4:** Evaluation of keyboard patterns features—paired *t*-test results for positive/negative opinions, *p*-values below 0.05 are marked bold.

Feature	*p*-Value (Rank)		
	**All Samples**	**Teacher Samples**	**Subject Samples**
	n=196	n=98	n=98
di_1D2D[0]	**0.0110** (5)	0.0862 (5)	0.0636 (5)
di_1D2D[1]	**0.0272** (7)	0.3060 (22)	**0.0406** (2)
di_1Dur[0]	0.9922 (51)	0.3446 (25)	0.3407 (25)
di_1Dur[1]	0.6492 (40)	0.9741 (51)	0.5640 (35)
di_1KeyLat[0]	**0.0092** (4)	**0.0365** (3)	0.1042 (10)
di_1KeyLat[1]	**0.0306** (8)	0.0964 (7)	0.1604 (16)
di_2Dur[0]	0.2982 (26)	0.2786 (21)	0.6908 (39)
di_2Dur[1]	0.8872 (47)	0.7062 (36)	0.5439 (34)
di_Dur[0]	**0.0456** (12)	0.2275 (14)	0.1146 (12)
di_Dur[1]	0.2168 (20)	0.3095 (23)	0.4593 (31)
di_NumEvents[0]	0.4972 (33)	0.2652 (20)	0.9736 (48)
di_NumEvents[1]	0.9639 (48)	0.7703 (40)	0.8601 (44)
tri_1D2D[0]	**0.0404** (9)	0.2257 (13)	0.1012 (9)
tri_1D2D[1]	0.0815 (16)	0.5219 (29)	0.0743 (6)
tri_1Dur[0]	0.8501 (46)	0.3832 (27)	0.2019 (20)
tri_1Dur[1]	0.4885 (31)	0.9490 (48)	0.3434 (26)
tri_1KeyLat[0]	**0.0425** (10)	0.1224 (8)	0.1821 (18)
tri_1KeyLat[1]	0.0679 (15)	0.2338 (16)	0.1717 (17)
tri_2D3D[0]	**0.0245** (6)	0.0953 (6)	0.1279 (14)
tri_2D3D[1]	0.0647 (14)	0.3188 (24)	0.1157 (13)
tri_2Dur[0]	0.2479 (22)	0.1326 (10)	0.8699 (45)
tri_2Dur[1]	0.3596 (28)	0.7236 (39)	0.3640 (27)
tri_2KeyLat[0]	**0.0085** (3)	**0.0297** (2)	0.1092 (11)
tri_2KeyLat[1]	**0.0448** (11)	0.1235 (9)	0.1894 (19)
tri_3Dur[0]	0.2469 (21)	0.2553 (19)	0.6115 (37)
tri_3Dur[1]	0.8431 (45)	0.6929 (35)	0.4735 (32)
tri_Dur[0]	0.0580 (13)	0.2302 (15)	0.1472 (15)
tri_Dur[1]	0.6020 (37)	0.8435 (43)	0.6057 (36)
tri_NumEvents[0]	0.5771 (35)	0.2485 (18)	0.8446 (43)
tri_NumEvents[1]	0.9901 (50)	0.6742 (34)	0.7637 (41)
SPACE	**0.0007** (1)	**0.0052** (1)	0.0556 (4)
BCKSPACE	0.1130 (18)	0.1398 (11)	0.4749 (33)
DEL	0.1119 (17)	0.1755 (12)	0.3223 (24)
UP	0.6051 (38)	0.5610 (32)	0.9763 (49)
DOWN	0.2587 (23)	0.2395 (17)	0.9767 (50)
LEFT	0.8165 (44)	0.7099 (38)	0.7818 (42)
RIGHT	0.3073 (27)	0.7091 (37)	**0.0382** (1)
SHIFT_L	0.9650 (49)	0.9601 (50)	0.8860 (46)
SHIFT_R	0.2980 (25)	0.5586 (31)	0.3935 (28)
L_di_1D2D[0]	0.2593 (24)	0.7766 (41)	0.0879 (7)
L_di_1D2D[1]	0.5596 (34)	0.6200 (33)	0.2197 (21)
L_di_Dur[0]	0.1976 (19)	0.8832 (45)	0.0949 (8)
L_di_Dur[1]	0.4886 (32)	0.9213 (47)	0.2769 (23)
L_first_shift_up	0.5827 (36)	0.5179 (28)	0.9156 (47)
R_di_1D2D[0]	0.4292 (30)	0.7910 (42)	0.4325 (29)
R_di_1D2D[1]	0.4109 (29)	0.8736 (44)	0.2713 (22)
R_di_Dur[0]	0.7049 (41)	0.9507 (49)	0.6509 (38)
R_di_Dur[1]	0.7665 (43)	0.9148 (46)	0.4554 (30)
R_first_shift_up	0.6406 (39)	0.5234 (30)	0.9910 (51)
CAPS	0.7135 (42)	0.3767 (26)	0.7256 (40)
SPEED	**0.0068** (2)	0.0738 (4)	**0.0439** (3)

**Table 5:** Evaluation of keyboard patterns features—mutual information measure.

Feature	Mutual Information				
	**Positive/Negative**			**High/Low**	
	**All Samples**	**Teacher Samples**	**Subject Samples**	**Pleasure**	**Arousal**
di_1D2D[0]	0.0714	0.0908	0.0236	0.0148	0.0528
di_1D2D[1]	0.0459	0.0000	0.0738	0.0228	0.0000
di_1Dur[0]	0.0842	0.0000	0.0316	0.0694	0.0000
di_1Dur[1]	0.0000	0.0923	0.0000	0.0592	0.0000
di_1KeyLat[0]	0.0869	0.1108	0.0181	0.0120	0.0186
di_1KeyLat[1]	0.0104	0.0044	0.1525	0.0000	0.0207
di_2Dur[0]	0.0000	0.0000	0.0119	0.0003	0.0116
di_2Dur[1]	0.0000	0.0500	0.0264	0.0000	0.0000
di_Dur[0]	0.0455	0.0035	0.0634	0.0071	0.0150
di_Dur[1]	0.0077	0.0000	0.0014	0.0000	0.0000
di_NumEvents[0]	0.0000	0.0737	0.0000	0.0100	0.0000
di_NumEvents[1]	0.0196	0.0543	0.0000	0.0466	0.0139
tri_1D2D[0]	0.0747	0.0000	0.1038	0.0437	0.0000
tri_1D2D[1]	0.0000	0.0000	0.0229	0.0000	0.0580
tri_1Dur[0]	0.0539	0.0000	0.0769	0.0363	0.0000
tri_1Dur[1]	0.0000	0.0731	0.0000	0.0323	0.0000
tri_1KeyLat[0]	0.0103	0.0000	0.0014	0.0000	0.0000
tri_1KeyLat[1]	0.0516	0.0547	0.0556	0.0000	0.0382
tri_2D3D[0]	0.0356	0.0000	0.0425	0.0202	0.0609
tri_2D3D[1]	0.0000	0.0045	0.0461	0.0000	0.0162
tri_2Dur[0]	0.0000	0.0128	0.0000	0.0000	0.0000
tri_2Dur[1]	0.0000	0.0429	0.0247	0.0000	0.0140
tri_2KeyLat[0]	0.0270	0.0677	0.0246	0.0000	0.0285
tri_2KeyLat[1]	0.0421	0.0000	0.1420	0.0000	0.0329
tri_3Dur[0]	0.0000	0.0000	0.0000	0.0000	0.0000
tri_3Dur[1]	0.0000	0.0408	0.0346	0.0094	0.0305
tri_Dur[0]	0.0494	0.0000	0.0286	0.0661	0.0000
tri_Dur[1]	0.0000	0.0000	0.0150	0.0456	0.0317
tri_NumEvents[0]	0.0000	0.0000	0.0000	0.0351	0.0575
tri_NumEvents[1]	0.0000	0.0562	0.0000	0.0136	0.0000
SPACE	0.0451	0.0773	0.0115	0.0137	0.0000
BCKSPACE	0.0000	0.0000	0.0548	0.0153	0.0004
DEL	0.0000	0.0000	0.0000	0.0516	0.0182
UP	0.0208	0.0000	0.0000	0.0018	0.0000
DOWN	0.0000	0.0000	0.0000	0.0578	0.0000
LEFT	0.0121	0.0206	0.0000	0.0315	0.0522
RIGHT	0.0000	0.0000	0.0000	0.0119	0.0506
SHIFT_L	0.0000	0.0000	0.0000	0.0535	0.0000
SHIFT_R	0.0195	0.0000	0.0000	0.0201	0.0521
L_di_1D2D[0]	0.0000	0.0000	0.0000	0.0018	0.0000
L_di_1D2D[1]	0.0126	0.1018	0.0705	0.0000	0.0000
L_di_Dur[0]	0.0513	0.0315	0.0413	0.0000	0.0324
L_di_Dur[1]	0.0000	0.0218	0.0000	0.0000	0.0226
L_first_shift_up	0.0103	0.0000	0.0000	0.0001	0.0000
R_di_1D2D[0]	0.0000	0.0000	0.0000	0.0000	0.0610
R_di_1D2D[1]	0.0000	0.0000	0.0000	0.0262	0.0001
R_di_Dur[0]	0.0000	0.0000	0.0329	0.0139	0.0102
R_di_Dur[1]	0.0000	0.0000	0.0000	0.0354	0.0000
R_first_shift_up	0.0000	0.0000	0.0000	0.0000	0.0000
CAPS	0.0264	0.0089	0.1154	0.0215	0.0000
SPEED	0.0504	0.1168	0.0000	0.0000	0.1211

**Table 6:** Testing the differences of PAD labels between the paired positive and negative opinions—results of two-sided Wilcoxon test.

	Pleasure	Arousal	Dominance
difference mean	−0.898	0.612	0.041
test statistic	230.5	709.5	658.0
*p*-value	0.0000	0.0009	0.9616

**Table 7:** Summary of classification accuracy for positive and negative opinions.

Data Set		Confusion Matrix		Precision	Recall	F1-Score
	Class	Positive	Negative			
	Positive	49	49	0.5632	0.5000	0.5297
Absolute	Negative	38	60	0.5505	0.6122	0.5797
	Average			0.5568	0.5561	0.5547
	Positive	57	41	0.6404	0.5816	0.6096
Relative	Negative	32	66	0.6168	0.6735	0.6439
	Average			0.6286	0.6276	0.6268

**Table 8:** Labels (class) distribution for different merging procedures.

	Number of Samples (High/Low)	
Labeling Procedure	Pleasure	Arousal
L1	163 (135/28)	146 (86/60)
L2	196 (135/61)	196 (86/110)
L3	196 (168/28)	196 (136/60)

**Table 9:** Summary of classification results for the level of of pleasure. The values marked bold are weighted averages of precision, recall and F1-score.

Data Set	Labeling		Confusion Matrix		Precision	Recall	F1-Score
		Class	High	Low			
	L1	High	97	38	0.8435	0.7185	0.7760
		Low	18	10	0.2083	0.3571	0.2632
		Average			**0.7344**	**0.6564**	**0.6873**
	L2	High	83	52	0.6803	0.6148	0.6459
Absolute		Low	39	22	0.2973	0.3607	0.3259
		Average			**0.5611**	**0.5357**	**0.5463**
	L3	High	136	32	0.8718	0.8095	0.8395
		Low	20	8	0.2000	0.2857	0.2353
		Average			**0.7758**	**0.7347**	**0.7532**
	L1	High	102	33	0.8361	0.7556	0.7938
		Low	20	8	0.1951	0.2857	0.2319
		Average			**0.7260**	**0.6748**	**0.6973**
	L2	High	101	34	0.7319	0.7481	0.7399
Relative		Low	37	24	0.4138	0.3934	0.4034
		Average			**0.6329**	**0.6378**	**0.6352**
	L3	High	143	25	0.8667	0.8512	0.8589
		Low	22	6	0.1935	0.2143	0.2034
		Average			**0.7705**	**0.7602**	**0.7652**

**Table 10:** Summary of classification results for the level of arousal. The values marked bold are weighted averages of precision, recall and F1-score.

Data Set	Labeling		Confusion Matrix		Precision	Recall	F1-Score
		Class	High	Low			
Absolute	L1	High	53	33	0.6235	0.6163	0.6199
		Low	32	28	0.4590	0.4667	0.4628
		Average			**0.5559**	**0.5548**	**0.5553**
	L3	High	93	43	0.6992	0.6838	0.6914
		Low	40	20	0.3175	0.3333	0.3252
		Average			**0.5824**	**0.5765**	**0.5793**
Relative	L1	High	64	22	0.6957	0.7442	0.7191
		Low	28	32	0.5926	0.5333	0.5614
		Average			**0.6533**	**0.6575**	**0.6543**
	L3	High	98	38	0.7538	0.7206	0.7368
		Low	32	28	0.4242	0.4667	0.4444
		Average			**0.6529**	**0.6429**	**0.6473**

## Data Availability

The data presented in this study are publicly available in a GitHub repository: https://github.com/agatakol/Keystroke-dynamics-patterns-while-writing-positive-and-negative-opinions.
